# First Principles Calculations on the Stoichiometric and Defective (101) Anatase Surface and Upon Hydrogen and H_2_Pc Adsorption: The Influence of Electronic Exchange and Correlation and of Basis Set Approximations

**DOI:** 10.3389/fchem.2019.00220

**Published:** 2019-04-16

**Authors:** Ruth Martínez-Casado, Milica Todorović, Giuseppe Mallia, Nicholas M. Harrison, Rubén Pérez

**Affiliations:** ^1^Departamento de Física de Materiales, Universidad Complutense de Madrid, Madrid, Spain; ^2^Departamento de Física Teórica de la Materia Condensada, Universidad Autónoma de Madrid, Madrid, Spain; ^3^Department of Applied Physics, Aalto University, Espoo, Finland; ^4^Department of Chemistry, Imperial College London, White City, London, United Kingdom; ^5^Condensed Matter Physics Center (IFIMAC), Universidad Autónoma de Madrid, Madrid, Spain

**Keywords:** density functional theory, oxides, anatase, hybrid functionals, defects, phthalocyanine

## Abstract

Anatase TiO_2_ provides photoactivity with high chemical stability at a reasonable cost. Different methods have been used to enhance its photocatalytic activity by creating band gap states through the introduction of oxygen vacancies, hydrogen impurities, or the adorption of phthalocyanines, which are usually employed as organic dyes in dye-sensitized solar cells. Predicting how these interactions affect the electronic structure of anatase requires an efficient and robust theory. In order to document the efficiency and accuracy of commonly used approaches we have considered two widely used implementations of density functional theory (DFT), namely the all-electron linear combination of atomic orbitals (AE–LCAO) and the pseudo-potential plane waves (PP–PW) approaches, to calculate the properties of the stoichiometric and defective anatase TiO_2_ (101) surface. Hybrid functionals, and in particular HSE, lead to a computed band gap in agreement with that measured by using UV adsorption spectroscopy. When using PBE+U, the gap is underestimated by 20 % but the computed position of defect induced gap states relative to the conduction band minimum (CBM) are found to be in good agreement with those calculated using hybrid functionals. These results allow us to conclude that hybrid functionals based on the use of AE–LCAO provide an efficient and robust approach for predicting trends in the band gap and the position of gap states in large model systems. We extend this analysis to surface adsorption and use the AE–LCAO approach with the hybrid functional HSED3 to study the adsorption of the phthalocyanine H_2_Pc on anatase (101). Our results suggest that H_2_Pc prefers to be adsorbed on the surface Ti_5*c*_ rows of anatase (101), in agreement with that seen in recent STM experiments on rutile (110).

## 1. Introduction

TiO_2_ is an important technological material with widespread applications in solar cells and photocatalysis (Fujishima and Honda, 1972; Fujishima et al., 2008; Schneider et al., 2014; Stetsovych et al., 2015). Although rutile is the stable bulk phase, anatase nanoparticles are often found to be the most active components in these applications.

Among the possible terminations, the (101) facet is found to be the most stable anatase surface (Labat et al., 2008) and represents a significant portion of the exposed surface area in the equilibrium crystallites that are characteristic of polycrystalline surfaces (Lazzeri et al., 2001; Barnard and Curtiss, 2005). The large anatase band gap (3.2 eV) limits its photocatalytic activity to the small ultraviolet portion of the solar spectrum (Hagfeldt and Grätzel, 1995). This has led to the use of a wide variety of approaches to engineer the band gap in order to enhance the activity. These include the incorporation of metallic and non-metallic ion impurities (Hoffmann et al., 1995). Hydrogen impurities are also usually introduced during processing and have significant influence on material properties, and therefore on the device performance. It is well established that hydrogen defects affect the ionic conductivity, electronic, and optical properties of both the bulk crystal and its surfaces (Amano et al., 2016). Hydrogen binds to lattice O^2−^ ions to form OH^−^ and this n-dopes the crystal modifying its electronic structure and, at elevated temperatures, proton transport makes a significant contribution to the ionic conductivity (Amano et al., 2016). Oxygen vacancies also play an important role in determining the surface chemistry and electronic properties of TiO_2_ (Morgan and Watson, 2010; Pan et al., 2013; Hongfei et al., 2015). Under typical processing conditions the dominant intrinsic defect is the oxygen vacancy, which also n-dopes the crystal and significantly affects photocatalytic properties (Liborio and Harrison, 2008). Previous DFT calculations have shown that TiO_2_ typically accommodates n-doping in localized states within the band gaps approximately 1eV below the CBM. These states could extend optical absorption to the visible region (Justicia et al., 2002; Liborio and Harrison, 2008). An alternative, and potentially complementary, approach is to enhance light absorption through surface species as happens in dye-sensitized solar cells (Graetzel, 2001). The details of the binding of the dye molecules to the surface are crucial for the improvement of device performance, with phthalocyanines being the most widely studied sysetm (Godlewski and Szymonski, 2013).

The quantitative analysis, and correct interpretation, of these processes has been assisted by the use of *ab initio* calculations of the surface structure and electronic properties. Anatase, as other reducible oxides, represents a challenge for standard implementations of DFT. Generalized gradient approximations (GGA) functionals often underestimate the band gap (Reshak et al., 2010) and provide a poor description of the localized gap states which are essentially lattice Ti^4+^(3*d*^0^) ions reduced to Ti^3+^(3*d*^1^) ions (Wang and Doren, 2005). It has been previously shown that the use of hybrid exchange functionals (where a proportion of Fock exchange is included in the exchange functional) provides a qualitatively correct description of the structure, energetics and electronic properties for many different materials, and in particular, for transition metal oxides (Patel et al., 2012, 2014; Sanches et al., 2014). However, in plane-wave codes, the use of hybrid exchange functionals increases dramatically the computational cost to an extent that prevents studies of complex geometries. For this reason, it is common to use the so-called DFT+U (Anisimov et al., 1993; Stetsovych et al., 2015) approximation, where LDA or GGA functionals are supplemented by an on-site local repulsion term U, representing the Coulombic self-interaction of the Ti 3*d* orbitals. This term partially corrects for the electronic self-interaction error inherent in local approximations to DFT and thus provides a more reliable, if somewhat *ad hoc*, description of the electron localization around Ti^3+^ ions.

In this work, firstly we document the performance of two widely employed DFT implementations (AE–LCAO and PP–PW) for computing the electronic structure of the defective (101) anatase surface using different exchange-correlation functionals. The defects considered are the dominant intrinsic ones for this system: single-atom oxygen sub-surface vacancies and hydrogen adsorbates (Setvin et al., 2013). The implementation of hybrid-exchange functionals using local atomic basis sets, in the CRYSTAL code (Dovesi et al., 2014), is very efficient, even when a high quality all-electron basis sets are used. CRYSTAL has therefore been used to investigate the stoichiometric and defective (101) anatase surface and upon hydrogen and H2Pc adsorption using the Perdew-Burke-Ernzerhof (PBE Perdew et al., 1996a) functional and the hybrid [PBE0 (Perdew et al., 1996b), B3LYP (Becke, 1988), and HSE (Heyd et al., 2003)] functionals. For the PP–PW calculations, only the PBE and PBE+U levels have been considered, rather than hybrid exchange functionals, due to the high computational cost of using hybrid functionals for systematic studies (Wu et al., 2009). In our experience, for PW calculations of the anatase bulk crystal, the computational time increases by three orders of magnitude when using hybrid exchange functionals compared to the AE–LCAO approximation. The comparison of the two approaches allows us to document the effects of the choice of basis set, the pseudopotential and the validity of the empirical PBE+U approach in this system. The PBE+U formalism has been shown to describe qualitatively correctly the defective rutile phase of TiO_2_, both in the bulk and on the surface, but the results for anatase surface remain ambiguous (Haa and Alexandrova, 2016). A similar analysis to the one presented here have been reported for bulk anatase (Finazzi et al., 2008; Valentin et al., 2009), but the complexity of the surface chemistry makes clear the value of the present study.

A judicious combination of the PP–PW and AE–LCAO approaches can provide an accurate description of complex defective and hybrid organic/oxide structures needed to understand and to improve the chemical reactivity and the photocatalytic properties. Once we have shown that the AE–LCAO approach with the HSE functional is able to accurately describe this surface, we go one step further by studying the adsorption of the phthalocyanine H_2_Pc on anatase (101). In the molecule-surface interaction, the effect of the Van der Waals forces is more important and has been taken into account by adding the D3 dispersion correction (HSED3) (Grimme and Krieg, 2010). We will show an analysis of the optimized structure and stability of this interaction, which will be a valuable tool in order to improve the efficiency of dye-sensitized solar cells. The article is structured as follows. In section 2 details are presented. In sections 3.1 and 3.2 the results for the bulk and stoichiometric anatase (101) surface are shown as a reference. The main features of the oxygen defective anatase (101) surface, together with the adsorption of both hydrogen and phthalocyanine on it are presented in sections 3.3, 3.4, and 3.5, respectively.

## 2. Computational Details

The calculations carried out with all-electron linear combination of atomic orbitals (AE–LCAO) have been performed using the CRYSTAL software (Dovesi et al., 2014). In the AE–LCAO formalism implemented in CRYSTAL (Dovesi et al., 2014), the crystalline orbitals are expanded as a linear combination of atom centered Gaussian orbitals with *s*, *p*, or *d* symmetry (the basis set). AE calculations were performed in which there is no shape approximation to the potential or density.

DFT has been applied with the PBE (Perdew et al., 1996a), PBE0 (Perdew et al., 1996b), B3LYP (Becke, 1988), and HSE (Heyd et al., 2003) functionals. The main numerical approximation in these calculations is the choice of the basis set (BS). In order to systematically approach the BS limit, a hierarchy of all-electron basis sets, labeled as BS1, BS2, and BS3 (see Table 1), has been selected for O, Ti. The most complete basis set (BS3), which includes extended polarization *d* orbitals for both the Ti and O atoms, has been used for the calculations reported below if not indicated. Apart from the obvious consequences on the structural and electronic properties, the subtle effect of the basis set on the description of the gap states will be discussed at the end of section 3.3. For the H_2_Pc molecule, we used BS4 in order to reduce the BS superposition error. In the H_2_Pc-anatase analysis we perform only AE–LCAO calculations with the HSE functional by adding the D3 dispersion correction (Grimme and Krieg, 2010), which is crucial for this kind of interactions.

**Table 1:** All-electron basis set hierarchy for Ti, O, H, N, and C.

	**Ti**	**O**	**H**	**N**	**C**
BS1	8-6411d(1)	8-611	3-1p(1)	—	—
BS2	8-6411d(11)	8-611	3-1p(1)	—	—
BS3	8-6411d(11)	8-611d(1)	3-1p(1)	—	—
BS4	8-6411d(311)	8-411d(11)	3-11p(1)	6-311d(1)	6-311d(1)

In the CRYSTAL (Dovesi et al., 2014) calculations, integration over the reciprocal space was carried out using Monkhorst-Pack (MP) meshes of 8 × 8 × 8 for the anatase bulk and 4 × 4 × 1 for the periodic slab representing the surface. The Coulomb and exchange series are summed directly in direct space and truncated using overlap criteria with thresholds of [7,7,7,7,14] (Pisani et al., 1988). The self-consistent field (SCF) algorithm was set to converge at the point at which the change in energy was < 10^−7^ Hartree per unit cell. Structural optimisation of both the bulk cell parameters and internal coordinates was performed using the Broyden-Fletcher-Goldfarb-Shanno scheme. Convergence was determined from the root-mean-square (rms) and the absolute value of the largest component of the forces. The thresholds for the maximum and the rms forces (the maximum and the rms atomic displacements) have been set to 0.00045 and 0.00030 (0.00180 and 0.0012) in atomic units. Geometry optimisation was terminated when all four conditions were satisfied simultaneously.

Calculations performed using the VASP code (Kresse and Furthmueller, 1996) include projected augmented wave (PAW) pseudopotentials (Bloechl, 1994; Kresse and Joubert, 1999) and a plane wave basis set with a cutoff of 500 eV. The SCF cycle stopping criterion was 10^−6^ eV. The PBE exchange-correlation functional was supplemented with an onsite U = 4eV terms on the Ti 3*d* orbitals to describe better the electronic structure of the (101) anatase surface and the electron localization on Ti^4+^ sites associated with the creation of its most common point defects. Ti PP is described by 4 valence electrons and O by 6 valence electrons. Previous studies on the anatase surface showed this U value provides the best description for the position of the defect-induced Ti gap states with respect to the conduction band minimum of TiO_2_ (Cheng and Selloni, 2009; Aschauer et al., 2010; Stetsovych et al., 2015). We included 15 Å of vacuum in order to isolate the two sides of the slab. Defect geometries were accessed using the robust Conjugate Gradient algorithm. Structural optimisation was halted when the value of the largest component of the forces reached a threshold value of 0.01 eV/Å. The Brillouin zone was sampled by a 6 × 6 × 2 MP mesh in bulk anatase calculations, and at Γ point in surface calculations in order to reduce the computational cost. Surface electronic properties were extracted from static calculations featuring a 4 × 4 × 1 MP mesh.

## 3. Results and Discussion

### 3.1. Bulk

**Figure 1:**
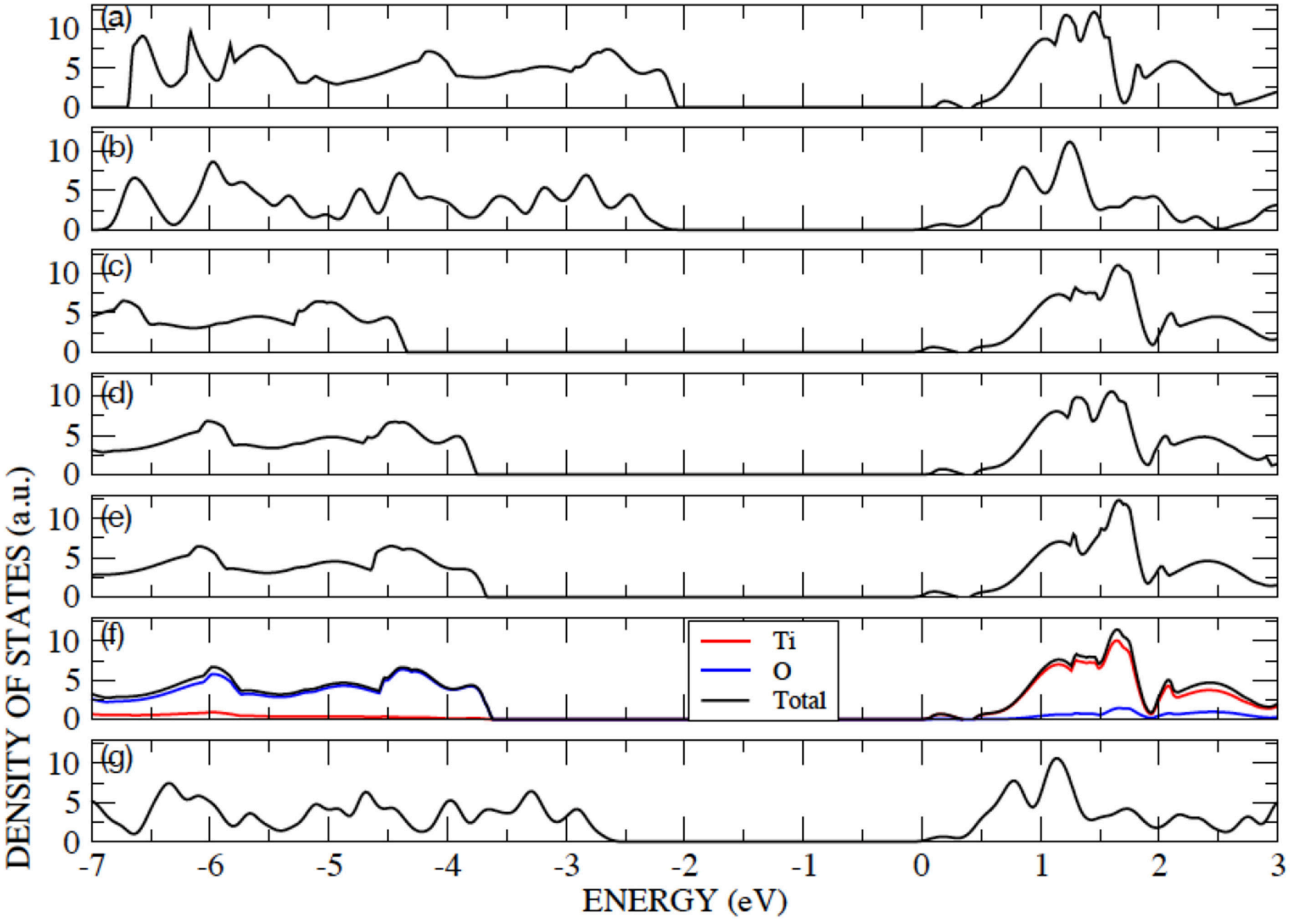
Color online) Density of states for bulk anatase performed with: **(a)** AE–LCAO (PBE), **(b)** PP–PW (PBE), **(c)** AE–LCAO (PBE0), **(d)** AE–LCAO (B3LYP), **(e)** AE–LCAO (HSED3) **(f)** AE–LCAO(HSE) and **(g)** PP–PW (PBE+U). Projected DOS is also shown for AE–LCAO (HSE).

The anatase structure belongs to the I4_1_/amd tetragonal space group and the unit cell is defined by the lengths of lattice vectors a and c and the oxygen internal coordinate u. The primitive cell contains two atoms in the asymmetric unit: a Ti ion at (0,0,0) and an O ion at (0,0,u), in fractional coordinates. Anatase is an indirect band-gap semiconductor with an optical band gap of approximately 3.2 eV (measured by using UV adsorption spectroscopy) (Reddy et al., 2003). Anatase bulk lattice parameters have been relaxed with AE–LCAO and the DFT functionals: PBE, PBE0, B3LYP, HSE and HSED3. The computed structures and eigenvalue gaps (here, referred to as band gaps for convenience) are presented in Table 2, and compared to the structure deduced from X-ray diffraction and the optical band gap measured using UV adsorption spectroscopy (Reddy et al., 2003). It is clear from Table 2 that the best agreement, for both lattice parameters and band gap, is obtained with the HSE functional. The results obtained within the HSED3 approach are also reported in Table 2, showing no significant changes with respect to pure HSE. The B3LYP lattice parameters and band gap are very similar to the ones previously obtained in the literature (Labat et al., 2008; Sanches et al., 2014). The eigenvalue gap is an estimate of the fundamental band gap. When using HSE and HSED3, the 13% overestimation of the optical band gap is not unreasonable as the optical gap is expected to be less than the fundamental gap due to the exciton binding energy. A comparison of the DOS calculated with PP–PW (PBE and PBE+U) and AE–LCAO (PBE, B3LYP and HSE) is presented in Figure 1. All DOS reported are plotted relative to the CBM to aid comparison. It is important to highlight the good agreement between AE–LCAO and PP–PW approaches with the same functional (PBE). Both methods provide a very similar band gap and lattice parameters suggesting that the PP–PW and local basis set approaches provide a very similar description of the electronic structure in both the valence and conduction bands. The projected density of states (DOS) for bulk anatase performed with AE–LCAO (HSE) shows that the conduction band is largely made up of Ti 3d orbitals and the upper valence band of O 2p orbitals, as one expects from a simple ionic model of the bonding.

**Table 2:** AE–LCAO and PP–PW relaxed lattice parameters and band gap for bulk anatase with respect to the experimental value measured by using X-ray diffraction and the adsorption spectrum (Reddy et al., 2003), respectively.

	**a(Å)**	**c(Å)**	**Indirect band gap (bulk) (eV)**
AE–LCAO PBE	3.803 (0.3 %)	9.770 (1.5 %)	2.12 (-33.7%)
PP–PW PBE	3.809 (0.5%)	9.724 (1.0%)	2.09 (-34.7%)
AE–LCAO PBE0	3.766 (-0.6 %)	9.655 (0.3 %)	4.26 (33.1%)
AE–LCAO B3LYP	3.790 (0.03 %)	9.776 (1.6 %)	3.77 (17.8%)
AE–LCAO HSE	3.766 (-0.6 %)	9.661 (0.4%)	3.63 (13.4%)
AE–LCAO HSED3	3.756 (-0.9 %)	9.522 (1.0%)	3.61 (12.8%)
PP–PW PBE+U	3.855 (1.7%)	9.842 (1.5%)	2.57 (-19.7%)
Observed (Reddy et al., 2003)	3.790	9.625	3.20

*The percentage error with respect to the experiment is reported in parenthesis*.

### 3.2. Stoichiometric (101) Surface

**Figure 2:**
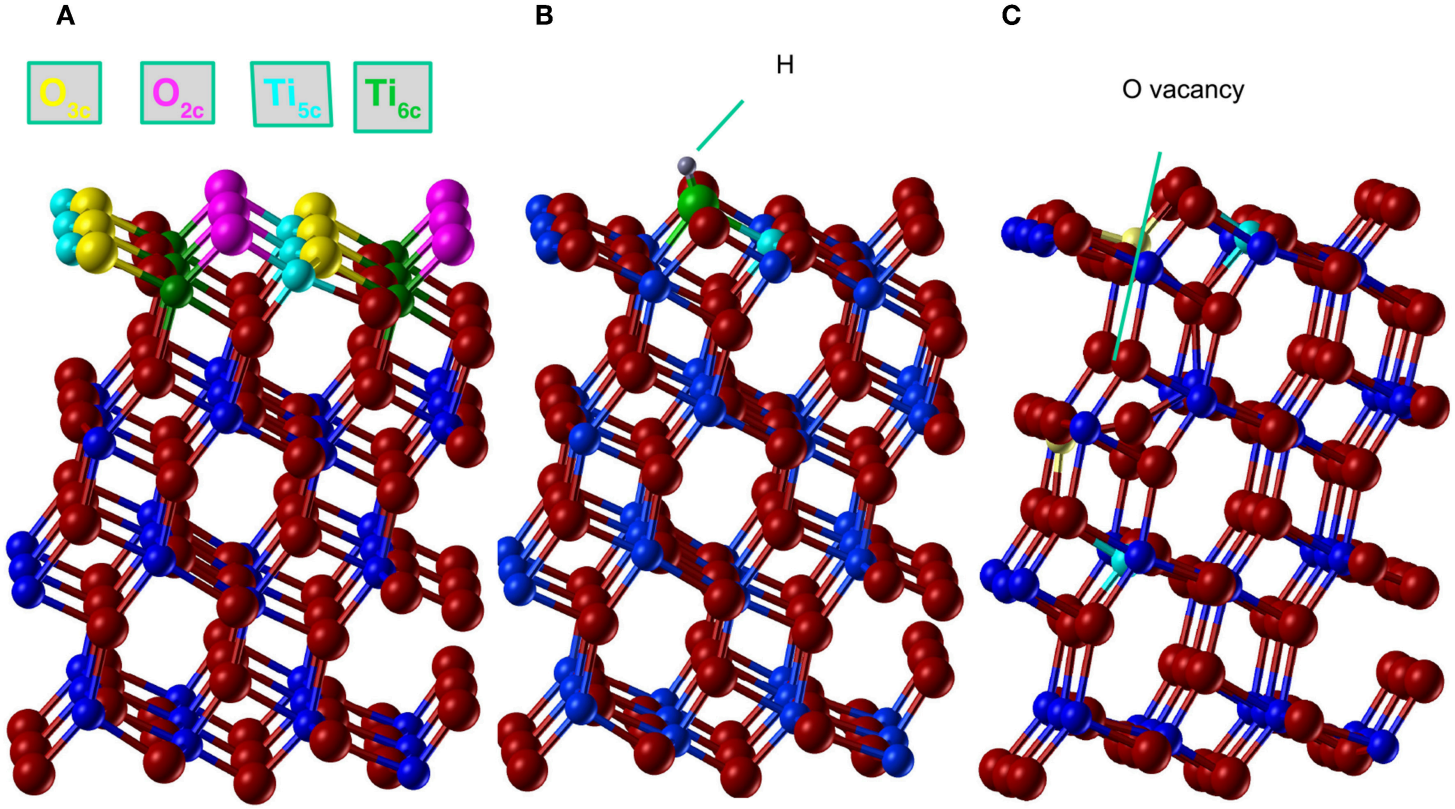
(Color online) Anatase (101) structure relaxed with AE–LCAO (HSE). **(A)** Stoichiometric surface, oxygen and titanium atoms are plotted in red and blue, respectively. The coordination of atoms in the top layer is represented in cyan (Ti_5*c*_), green (Ti_6*c*_), magenta (O_2*c*_), and yellow (O_3*c*_). **(B)** H adsorption, where H atom is represented in gray, the O_2*c*_ bonded to H in green and the spin polarized Ti_5*c*_ in cyan, **(C)** O monovacancy, where the spin polarized Ti_5*c*_ atoms are represented in cyan (minimum energy configuration) and yellow (alternative configuration of the spin).

The (101) surface has two possible terminations, both resulting in stoichiometric, nonpolar surfaces. In this study we consider the low-energy termination (Sanches et al., 2014), where, as shown in Figure 2A, one of each of the following atoms are exposed per periodic cell: O_2*c*_,O_3*c*_,Ti_5*c*_ and Ti_6*c*_ producing the characteristic saw-tooth (101) surface structure. The 2c, 3c, 5c, and 6c indexes represent the two, three, 5 and 6 fold–coordinated atoms, respectively. The surface was modeled with a 2D periodic slab that includes four TiO_2_ trilayers with the in–plane periodicity given by a 1 × 3 supercell of the conventional surface cell.

The fact that both the AE–LCAO and PP–PW approaches provide a very similar description at the PBE level suggests that the plane-wave and local Gaussian orbital basis sets used are both well converged and that the pseudopotentials are describing the valence-core interactions adequately. This also facilitates the comparison of the results of PP–PW-PBE+U calculations with AE–LCAO-HSE and -B3LYP calculations, as we can be confident that the numerical approximations used are equivalent. This allows us to assess the accuracy of the widely used DFT+U approach.

**Figure 3:**
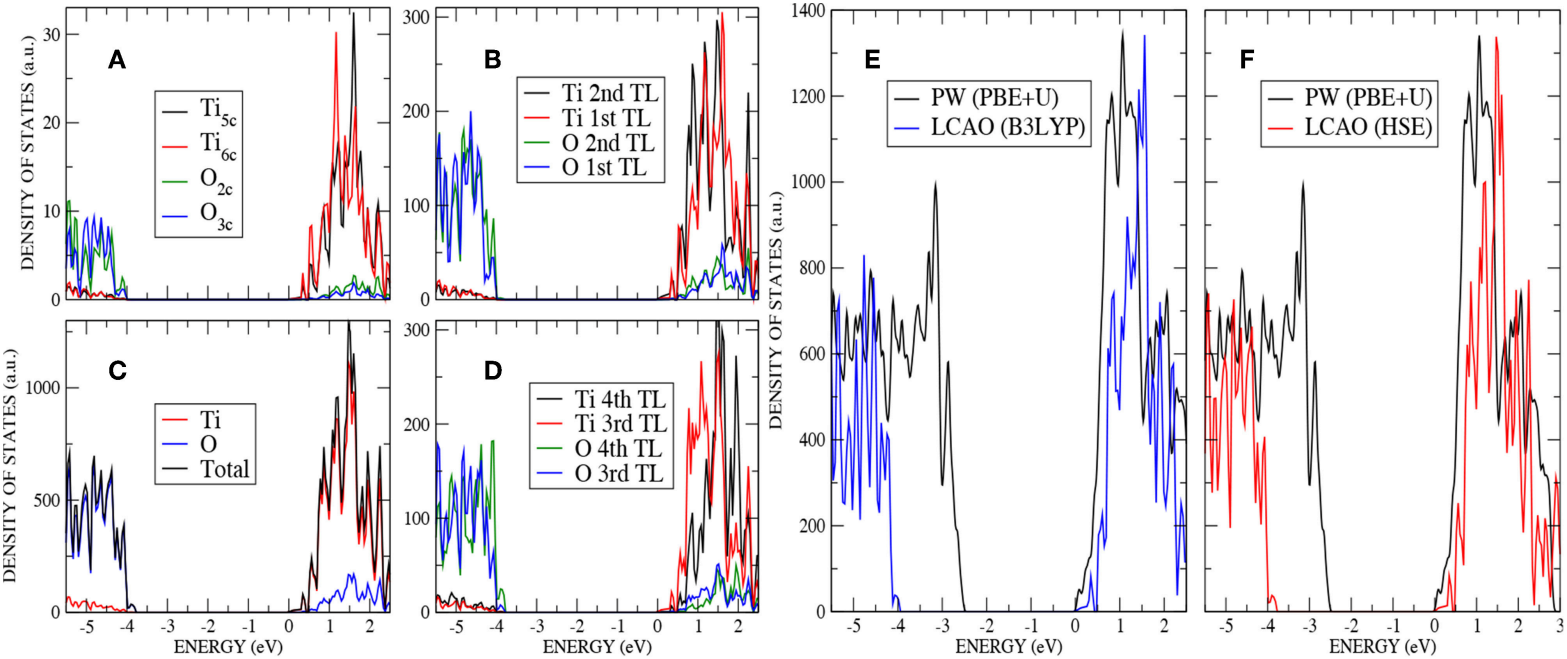
(Color online) Stoichiometric anatase (101) : **(A)** PDOS on the most important atoms (O_2*c*_,O_3*c*_,Ti_5*c*_ and Ti_6*c*_) of the top layer by using AE–LCAO (HSE), **(B)** PDOS on the two top trilayers by using AE–LCAO (HSE), **(C)** DOS by using AE–LCAO (HSE), **(D)** PDOS on the two bottom trilayers by using AE–LCAO (HSE) (bulk-like region), **(E)** DOS comparison of AE–LCAO (B3LYP) and PP–PW (PBE+U), and **(F)** DOS comparison of AE–LCAO (HSE) and PP–PW (PBE+U).

An analysis of the DOS for the stoichiometric anatase (101) calculated with AE–LCAO and HSE (Figure 3) exhibits a similar structure to that seen in the bulk crystal. The conduction band is mainly composed of Ti 3d orbitals and the upper valence band of O 2p orbitals. In order to separate the surface contribution from that of the bulk, the DOS have been plotted in four different ways: projected (PDOS) on the most important atoms of the top layer (O_2*c*_, O_3*c*_, Ti_5*c*_, and Ti_6*c*_) (Figure 3A), PDOS on the two top trilayers (Figure 3B), total DOS (Figure 3C), and PDOS on the two bottom trilayers (Figure 3D).

The computed DOS of the stoichiometric surface using the PP–PW (PBE+U) and AE–LCAO (HSE and B3LYP) approximations are compared in Figure 3. It can be seen that the global hybrid (B3LYP) and screened exchange (HSE) approaches produce very similar band width, shape and band gap. The empirical PBE+U method yields similar band widths but a significantly smaller band gap.

In Table 3 the bond distances and atom height (with respect to the position of the first oxygen atom of the second trilayer) using PP–PW (PBE+U) and AE–LCAO (HSE and B3LYP) are reported for the selected atoms of the surface layer. The surface structure is found to be relatively insensitive to both the numerical approximation and to the exchange correlation functional used.

**Table 3:** Bond distance and height of relevant atoms in the top layer (Å) for AE–LCAO and PP–PW in the anatase (101) stoichiometric surface, hydrogen adsorption and oxygen monovacancy.

	**Ti_**5*c***_-O_**2*c***_**	**Ti_**6*c***_-O_**2*c***_**	**H-O_**2*c***_**	**Ti_**5*c***_ height**	**Ti_**6*c***_ height**	**O_**2*c***_ height**	**H height**
**PP–PW(PBE+U)**							
stoichiometric	1.86	1.90	—	2.64	2.01	3.48	—
Hads	2.07	2.05	0.97	2.72	1.87	3.55	4.46
Ovac	2.04	1.76	—	2.71	2.5	3.81	—
**AE–LCAO(B3LYP)**							
stoichiometric	1.83	1.84	—	2.67	2.13	3.54	—
Hads	2.02	2.05	0.96	2.66	1.91	3.57	4.26
Ovac	2.02	1.72	—	2.75	2.54	3.83	—
**AE–LCAO(HSE)**							
stoichiometric	1.83	1.82	—	2.64	2.1	3.50	—
Hads	2.0	2.03	0.96	2.64	1.91	3.54	4.33
Ovac	2.01	1.71	—	2.73	2.53	3.81	—

*The height of the atoms is defined with respect to the position of the first oxygen atom of the second trilayer in order to allow a direct comparison*.

### 3.3. Oxygen Vacancy on (101) Anatase

A simple model of the reduced anatase surface can be generated by oxygen desorption induced by heating. Although, as the least coordinated, the outermost O_2*c*_ oxygen atoms might be expected to be the first to leave the surface, previous work (Setvin et al., 2013) has shown that these surface vacancies are unstable and quickly evolve to form subsurface vacancies. We therefore consider a subsurface oxygen vacancy model where an oxygen atom is removed from the second trilayer of the surface (see Figure 2C).

**Figure 4:**
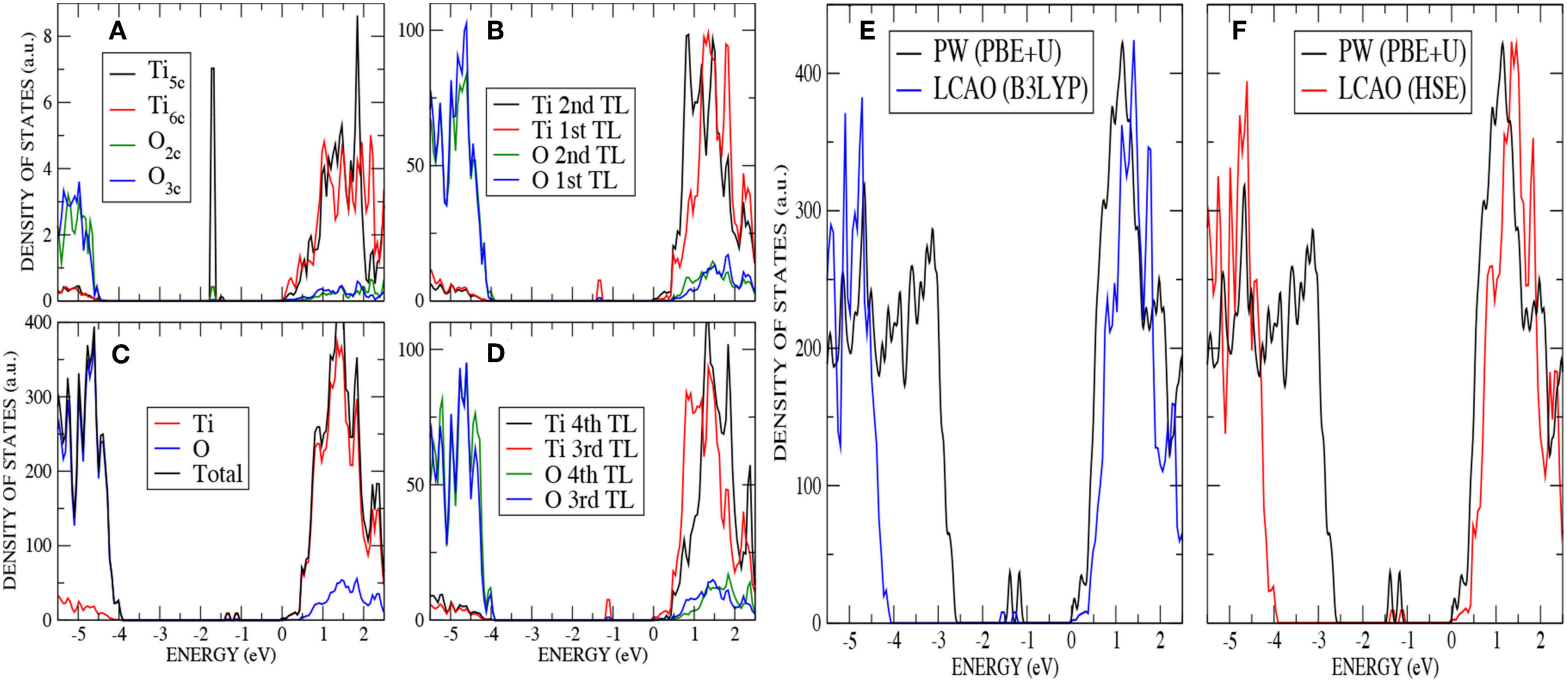
(Color online) O monovacancy on anatase (101) : **(A)** PDOS on the most important atoms (O_2*c*_,O_3*c*_,Ti_5*c*_, and Ti_6*c*_) of the outermost layer by using AE–LCAO (HSE), **(B)** PDOS on the two top trilayers by using AE–LCAO (HSE), **(C)** DOS by using AE–LCAO (HSE), **(D)** PDOS on the two bottom trilayers by using AE–LCAO (HSE) (bulk-like region), **(E)** DOS comparison of AE–LCAO (B3LYP) and PP–PW (PBE+U), and **(F)** DOS comparison of AE–LCAO (HSE) and PW (PBE+U).

The extra two electrons left after the vacancy formation could form an F-center at the vacant lattice site or enter the Ti 3*d* band and, potentially, localize to form Ti^3+^ species. A number of previous theoretical studies have described the formation of these localized states (Lindan et al., 1997; Muscat et al., 1999; Chen et al., 2001; Deak et al., 2015). The ground state considered here corresponds to the occupation of a localized 3*d* orbital on each of two next-nearest neighbors, a Ti_5*c*_ atom in the first trilayer and a Ti_6*c*_ in the third trilayer (cyan atoms in Figure 2C). A previous PP–PW (PBE+U) study also suggested that this configuration for the reduced titanium ions is the most stable (Stetsovych et al., 2015). This is also the case in calculations based on hybrid functionals. For example, the configuration where two nearest neighbor Ti ions are reduced (magenta atoms in Figure 2C) is a local energy minimum but has an energy 0.82 eV higher than the one described above. The reduction of two Ti^4+^ ions is confirmed by the spin–polarized DOS presented in Figure 4. The two Ti^3+^ ions have a magnetic moment of 0.90 and 0.91 μ_*B*_ for AE–LCAO (3LYP), 0.92 and 0.91 μ_*B*_ for AE–LCAO (HSE) and 0.91 and 0.89 μ_*B*_ for PP–PW (PBE+U). The new localized states, mainly formed by Ti 3*d* orbitals from the Ti_6*c*_ in the third trilayer and the Ti_5*c*_ in the first trilayer, appear at 1.0 and 1.3 eV below the conduction band for AE–LCAO (HSE). PP–PW (PBE+U) gives a very similar result (1.1, 1.3 eV), while AE–LCAO–B3LYP predicts those states to be deeper in the band gap (1.2, 1.5 eV). The AE–LCAO (HSE) calculation is in good agreement with photoemission experiments, which identify a state at around 1 eV bellow the CBM (Thomas et al., 2007).

The relaxed structure for the subsurface vacancy can be seen in Figure 2C, where the Ti^3+^ ions depicted in cyan. The charge localization has a profound influence on the structure, as shown by the bond distances and relative height of the key atoms in the top layer in Table 3. A significant decrease of the Ti_6*c*_-O_2*c*_ bond is seen. The Ti${}_{6c}^{3+}$-O_2*c*_ bond is reduced by 0.1 Å, with respect to the Ti${}_{6c}^{4+}$-O_2*c*_ bond. There are also significant relaxations in the reduced top Ti_6*c*_, that changes its coordination from 6 to 5 and moves up by 0.5 Å. In the presence of two polarons and a vacancy, the polaronic distortions might interact and therefore affect the total energy, geometry and position of the gap state. This structural feature appears consistently in all of the calculations presented here and can thus be considered to be insensitive to the approximation of the electronic exchange and correlation.

Previous combined STM/AFM experiments show a strong distortion of the O_2*c*_ site above the vacancy which is lifted up by 0.3 Å (Stetsovych et al., 2015). PP–PW (PBE+U) and AE–LCAO (B3LYP) support this result with the O_2*c*_ moving up by 0.28 and 0.31 Å, respectively. AE–LCAO (HSE) reproduces this trend with a normal displacement of 0.38 Å, identical to the PP–PW–PBE+U result.

**Figure 5:**
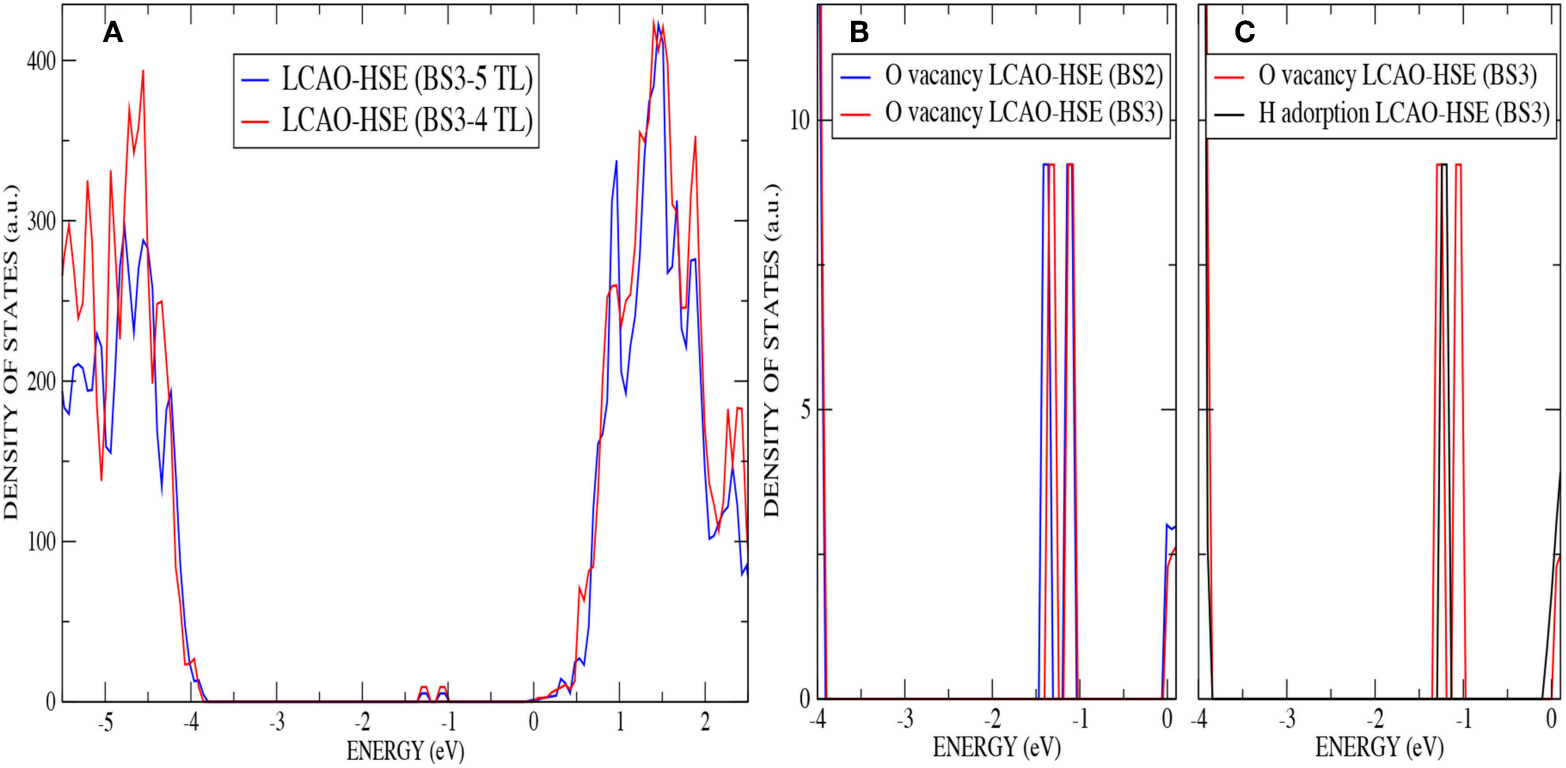
(Color online) **(A)** DOS comparison for the oxygen monovacancy on anatase (101) by using AE–LCAO (HSE) with four and five trilayers **(B)** Band gap for the oxygen monovacancy on anatase (101) by using AE–LCAO (HSE) with BS2 and BS3. **(C)** Band gap for an hydrogen adsorption and oxygen monovacancy on anatase (101) by using AE–LCAO (HSE) with BS3.

The four–layer slab used so far is clearly suitable to describe the relaxations in the top layers associated with the creation of the surface and the H–defect. In the case of the subsurface vacancy, where one of the reduced Ti^3+^ ions is located in the third trilayer, one may wonder if an additional trilayer is needed in order to relax the local strain induced by the larger ion, as the fourth trilayer has been kept fixed. This strain can be more easily accommodated by atomic relaxations when the reduced ion is located in the first trilayer. This is consistent with the fact that the gap states associated with the two Ti^3+^ ions have different energy, the one associated with the Ti^3+^ in the top trilayer being lowered in energy. Thus, we have optimized the structure of a five–trilayer slab using AE–LCAO–HSE and keeping fixed the fifth (bottom most) trilayer. The structural analysis shows no significant differences, with very similar bond distances among the key atoms and the same surface relaxation pattern, where the O_2*c*_ above the vacancy moves up again by 0.4 Å. Regarding the electronic properties, a comparison of the DOS calculated with four and five trilayers (see Figure 5A) shows that they are nearly identical. The position of the new localized states and local spin magnetic moment (0.94 and 0.93 μ_*B*_) are also very similar to the ones calculated with four trilayers. These results confirm that when the donated electrons are accommodated in the described positions, a four–trilayer slab provides an adequate description.

The localized states associated with the Ti^3+^ ions provide a rigorous test of the flexibility of the local Gaussian basis sets used. The nature of the localized d-state is very different to that of the delocalized d-band of the undoped system. We therefore expect some sensitivity to the flexibility of the basis set used to describe the Ti-d derived orbitals. In Figure 5B the DOS in the band gap is displayed for both the oxygen subsurface vacancy with the two basis sets, BS2 and BS3. Although the calculations using BS2 reproduce the surface structure very accurately, the computed position of the defects state relative to the CBM differs by 6% to that predicted by the more flexible BS3. The computational time increases by 33% by using BS3 instead of BS2.

In Table 4, AE–LCAO (B3LYP and HSE) calculations show an increase of the surface band gap of around 2% when an oxygen monovacancy is present. This result can be due again to the approximation of considering few layers to simulate the real surface or to the interaction of polaronic distortions as explained above. AE–LCAO (HSE) also describes very similar peaks for the gap state corresponding to the Ti^3+^ ion on the outermost layer in both defects, as it can be seen in Figure 5C.

**Table 4:** Band gap for the stoichiometric anatase (101) and the defective surface by using AE–LCAO and PP–PW.

	**Stoichiometric (eV)**	**H adsorption (eV)**	**O vacancy (eV)**
AE–LCAO B3LYP	3.93	3.86	4.0
AE–LCAO HSE	3.77	3.70	3.83
PP–PW PBE+U	2.41	2.41	2.41

The heterogeneous photocatalysis is based on the ability of photocatalysts to absorb the light energy required to generate electron–hole pairs for a surface reaction. TiO_2_ can only absorb ultraviolet (UV) light, but its optical properties can be enhanced by defect engineering. Both oxygen vacancies and hydrogen adsorption give rise to local states below the CB edge, which can extend the light absorption of TiO_2_ from the UV to the visible ranges. The formation energy is calculated by using the formula:

(1) $$\begin{array}{r} E_{f}=E_{def}+1/2E_{O2}-E_{nodef}, \end{array}$$

where *E*_*def*_ and *E*_*nodef*_ are the energies of the defective and non defective anatase(101), respectively, and *E*_*O*2_ is the energy of the oxygen molecule. The calculated formation energy with AE–LCAO (HSE) is 2.6 eV, in good agreement with the value 2.9 eV, given in the literature (Haa and Alexandrova, 2016).

### 3.4. H Adsorption on (101) Anatase

The adsorption mechanism for H investigated here corresponds to the R arrangement in the DFT study by Leconte et al. (2002), since this was found to be the most favorable adsorption mode. A hydrogen atom is adsorbed onto a surface oxygen atom, upon which a reduction of a nearby titanium atom takes place. In our calculation, a neutral H atom is adsorbed to O^2−^ resulting in the formation of OH^−^ and the donation of an electron to the surface CB.

This electron is transferred to the oxide lattice, and occupies one of the localized 3*d* orbitals of the nearest neighbor Ti_5*c*_ ions. As a result, this Ti^4+^ ion is reduced and converted to a Ti^3+^ ion. The analysis of the spin (unpaired electrons) population confirms the transfer of an electron onto the Ti ion. The magnetic moment is 0.920 μ_*B*_ for PP–PW (PBE+U), 0.957 μ_*B*_ for AE–LCAO (HSE) and 0.944 μ_*B*_ for AE–LCAO (B3LYP). This localization of the electron is due to on–site electronic correlation and therefore one could expect it to be much less when compared to the LDA or GGA approximations.

In Figure 2B, the final relaxed structure using the AE–LCAO method and the HSE functional is displayed. The bond distances and atom heights for the key atoms in the top layer calculated with different exchange-correlation functionals are compared in Table 3. All the functionals consistently predict the O_2*c*_ atom bonded to H that moves outwards by 0.13 Å. The height of the H atom above the O_2*c*_ plane is directly observable in non–contact atomic force microscopy (NCAFM) images (Stetsovych et al., 2015) resulting in a value of 0.11 Å, which is in good agreement with the one predicted here.

**Figure 6:**
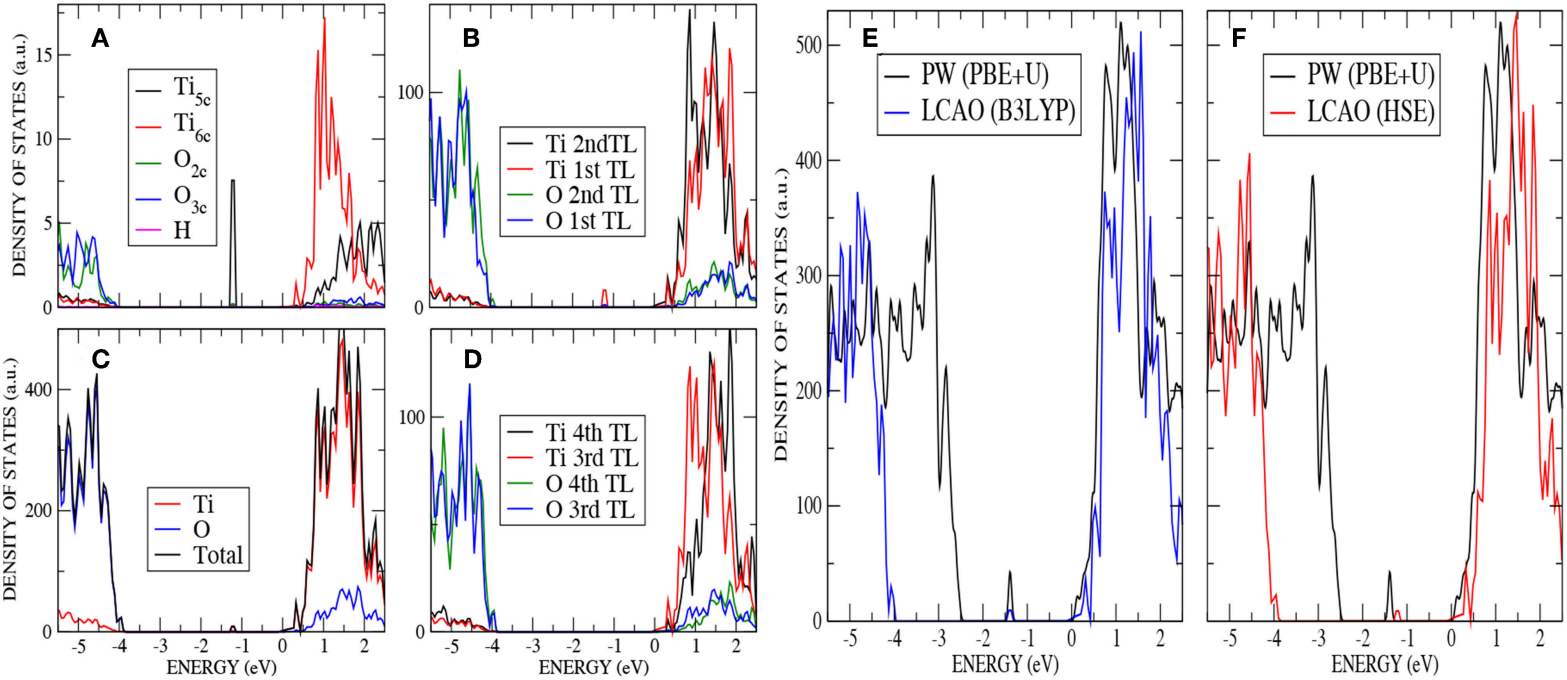
(Color online) H adsorption on anatase (101) : **(A)** PDOS on the most important atoms (O_2*c*_,O_3*c*_,Ti_5*c*_, and Ti_6*c*_) of the top layer by using AE–LCAO (HSE), **(B)** PDOS on the two top trilayers by using AE–LCAO (HSE), **(C)** DOS by using AE–LCAO (HSE), **(D)** PDOS on the two bottom trilayers by using AE–LCAO (HSE) (bulk-like region), **(E)** DOS comparison of AE–LCAO (B3LYP) and PP–PW (PBE+U), and **(F)** DOS comparison of AE–LCAO (HSE) and PP–PW (PBE+U).

The calculated DOS displayed in Figure 6 –analogous to Figure 3 for the stoichiometric surface– shows the presence of a localized state 1.2 eV below the conduction band minimum (CBM) for AE–LCAO with HSE (and 1.4 eV for AE–LCAO with B3LYP and PP–PW with PBE+U). This new peak in the band gap, mainly formed by Ti 3*d* states coming from the CBM, is the signature of the reduction of one Ti^4+^ ion of the surface layer to Ti^3+^. This behavior has been observed in UV absorption spectra and documented in previous calculations of the K atom adsorbed on TiO_2_ surface, where the donation of the electron to TiO_2_ was localized in a similar state (Muscat et al., 1999). It is remarkable that, in spite of the different position of the valence band maximum, PBE+U predicts a position of that peak with respect to the CBM in agreement with hybrid functionals. In previous infrared spectroscopy experiments and theoretical calculation based on GGA+U on rutile reduced by hydrogen, these gap states have been reported to be in the range 0.75-1.18 eV below the CBM (Cronemeyer, 1959; Finazzi et al., 2008; Islam et al., 2011; Antila et al., 2015), which is consistent with the data presented here. Therefore, both the AE–LCAO and PP–PW implementations predict a similar position of the band gap state due to the localization of the extra electron on the 3d orbital of a Ti_5*c*_ of the top layer.

It has been stated before that the introduction of defects on TiO_2_ can lead to a modification of the band gap value (Naldoni et al., 2012; Mehta et al., 2016). In Table 4, a comparison of the computed electronic structure of the stoichiometric and defective surface is presented and the band gap is indicated. The adsorption of hydrogen on anatase (101) leads to a small reduction of the band gap of around 2 % in both the HSE and B3LYP approximations (this result agrees with the value of 1.8% found by diffusion reflectance spectroscopy Mehta et al., 2016). In calculations based on PBE+U the inclusion of defects produces no change in the band gap.

The formation energy is calculated by using the formula:

(2) $$\begin{array}{r} E_{f}=E_{slab}+E_{H2}/2-E_{H/slab}, \end{array}$$

where *E*_*slab*_ and *E*_*H*/*slab*_ are the energies of the slabs without and with hydrogen adsorbed on the surface, respectively, and *E*_*H*_ is the energy of atomic hydrogen. The calculated formation energy with AE–LCAO (HSE) is 2.26 eV, in good agreement with the value 2.31 eV, given in the literature (Islam et al., 2011).

### 3.5. Adsorption of Unsubstituted Phthalocyanine on Anatase(101)

**Figure 7:**
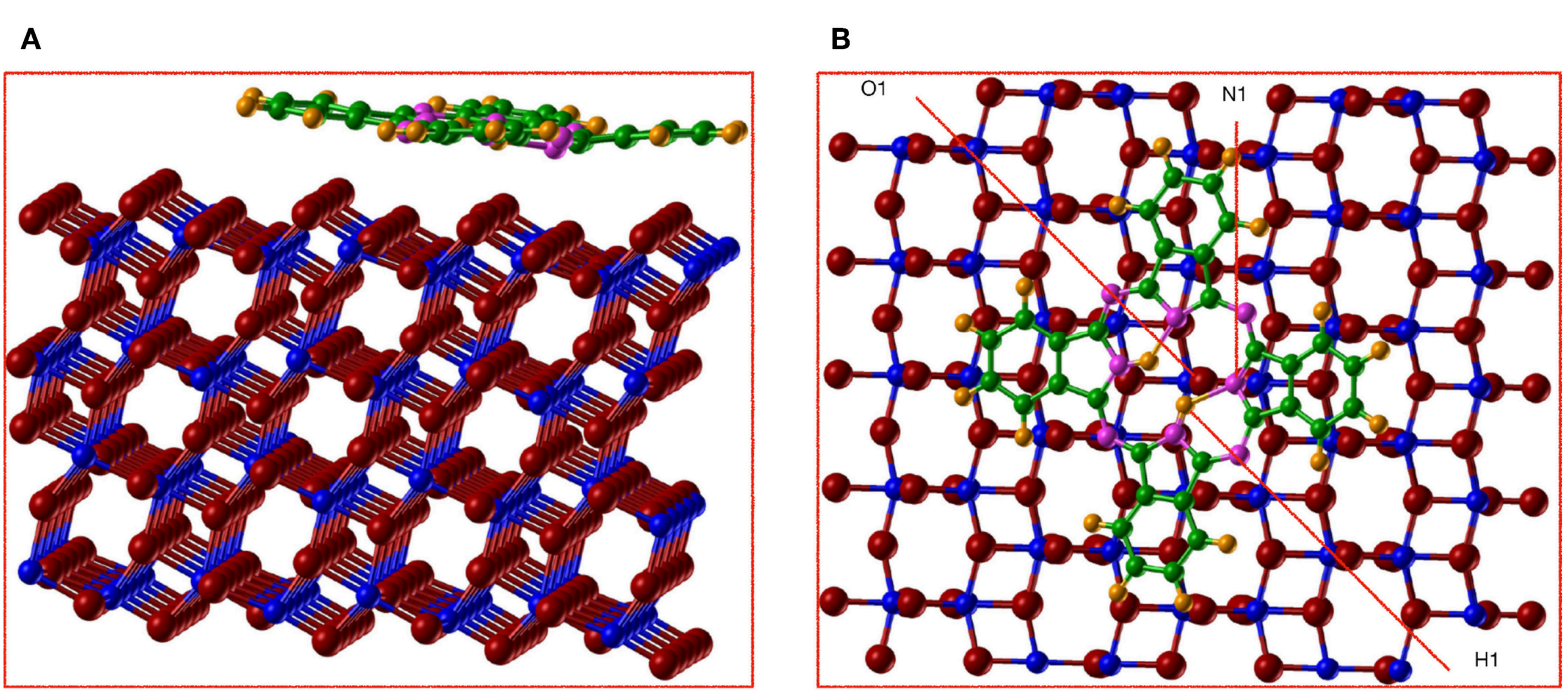
(Color online) **(A)** side view and **(B)** top view of the optimized structure for the adsorption of H_2_Pc on anatase (101) by using AE–LCAO (HSE) and centering the molecule on the O_2*c*_ row. We represent Ti in blue, O in red, N in magenta, C in green, and H in orange.

**Figure 8:**
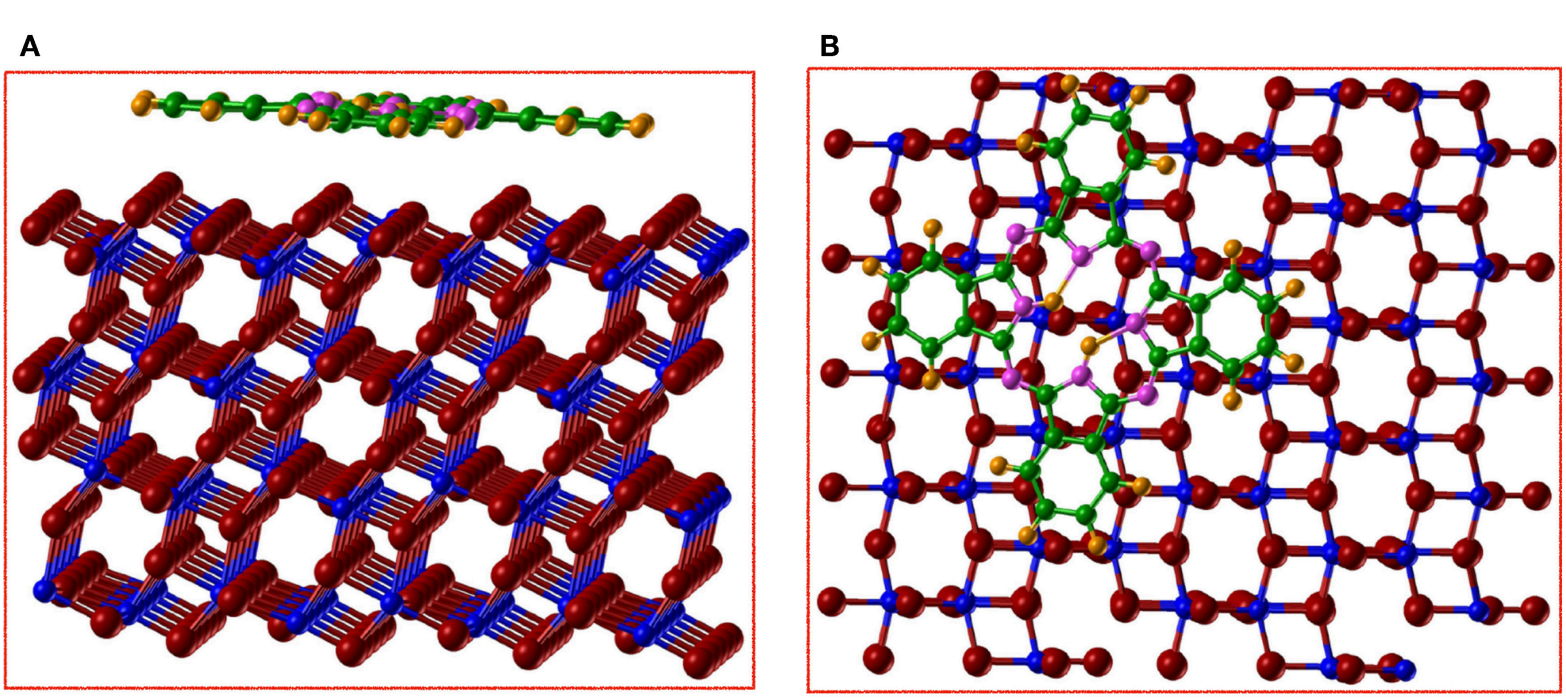
(Color online) **(A)** side view and **(B)** top view of the optimized structure for the adsorption of H_2_Pc on anatase (101) by using AE–LCAO (HSE) and centering the molecule on the Ti_5*c*_ row. We represent Ti in blue, O in red, N in magenta, C in green, and H in orange.

The data presented above suggested that the AE–LCAO-HSE approach is generally the most satisfactory for describing the electronic structure of the stoichiometric and reduced anatase surface. We therefore use this approach to consider the adsorption of H_2_Pc on anatase (101). In this calculation we also include the effects of London dispersion interactions through the empirical Grimme D3 correction, as these are likely to play a role in the molecule-surface binding. Initial geometries with the molecule placed centered on O_2*c*_ or Ti_5*c*_ rows of the anatase surface have been used in order to explore alternative binding sites on the anatase surface. The optimized structures for both adsorption sites are shown in Figures 7, 8. Local minima are found at both sites. It is perhaps surprising to observe the significant changes in the molecular structure of H_2_Pc induced when it is centered on O_2*c*_. The molecule becomes non–co–planar and the C-H rings move away from the surface by 0.3–0.4 Å with respect to the central atoms of the molecule, which makes clear the strong repulsion between these rings and the O_2*c*_. The reason for this behavior can be the strength of the C-H bond which makes it relatively unreactive. However, the N atoms from the molecule interact strongly with the surface Ti atom, resulting in the N atom placed just on top of the Ti_5*c*_ (N1 in Figure 7B) moving closer to the surface by 0.3 Å, with respect to the rest of the N in the molecule.

The central hydrogens of the molecule form a hydrogen bond of 1.8 Å with the neighboring nitrogens, while one of them (H1) tries to attach to the O_2*c*_ of the surface. These effects make the O_2*c*_ (O1) to move up by 0.1 Å. On the contrary when the molecule is centered on Ti_5*c*_ (Figure 8), its remains relatively flat with just variations of 0.1 Å in height between the different atoms. For both sites, H_2_Pc is adsorbed with a board altitude that reaches approximately 2.7 Å, in agreement with the result obtained by using STM on rutile (Godlewski and Szymonski, 2013). The calculated adsorption energy is of –3.18 eV with the molecule centered on the oxygen row and of −3.54 eV with the molecule centered on the titanium row. Therefore, the adsorption of H_2_Pc is more favorable if the molecule is centered on Ti_5*c*_ by 0.36 eV. It is clear that when the molecule is adsorbed centered in the O_2*c*_ the repulsion between the surface and the C-H rings is stronger than the bonds formed.

## 4. Conclusions

The AE–LCAO approach is a robust and efficient ab initio method to correctly describe the defects and adsorption of molecules on oxide surfaces. In this work, we have documented the performance of two widely used methods based on Density Functional Theory: linear combination of atomic orbitals (AE–LCAO) and plane waves (PP–PW) to describe the properties of the stoichiometric and defective TiO_2_(110) anatase surface. Full relaxation of stoichiometric and defective anatase (101) surfaces has been carried out using the PBE functional and the hybrid PBE0, B3LYP and HSE functionals. For the PP–PW calculations, only the PBE and PBE+U levels of theory have been considered due to the high computational cost of using hybrid functionals for systematic studies. Hybrid functionals, and, in particular, HSE, lead to a computed band gap in excellent agreement with the experimental data. PBE+U underestimates the gap by 20 % but the predicted gap states induced by the defect with respect to the bottom of the conducting band are in good agreement with the hybrid functionals. An understanding of the defective anatase surface is very important for many technological applications. It has been argued that vacancies are very important in the photocatalytic property of surfaces. The formation of mid-gap states, as the ones described here, could improve the photoactivity of the system in the visible light region. Once we have proved that the AE–LCAO approach with the HSE functional is able to accurately describe this surface, we went one step further by studying the adsorption of the phthalocyanine H_2_Pc on anatase (101). H_2_Pc was placed both on top of the oxygen and titanium rows of anatase (101), with the latter lower in energy by 0.36 eV. This work presents an ab initio study of the adsorption of H_2_Pc on anatase (101), and makes clear the influence of the adsorption site in the phthalocyanine-anatase interaction. The AE–LCAO method presented here opens a way to correctly describe the defects and adsorption of molecules on oxide surfaces by using hybrid exchange functionals at a reasonable computational cost, which will be a valuable tool in order to improve the efficiency of dye-sensitized solar cells.

## Author Contributions

All authors listed have made a substantial, direct and intellectual contribution to the work, and approved it for publication.

### Conflict of Interest Statement

The authors declare that the research was conducted in the absence of any commercial or financial relationships that could be construed as a potential conflict of interest.
